# Rickets guidance: part I—diagnostic workup

**DOI:** 10.1007/s00467-021-05328-w

**Published:** 2021-12-15

**Authors:** Dieter Haffner, Maren Leifheit-Nestler, Andrea Grund, Dirk Schnabel

**Affiliations:** 1 Department of Pediatric Kidney, Liver and Metabolic Diseases, Hannover Medical School, Carl-Neuberg-Str. 1, 30625 Hannover, Germany; 2 Hannover Medical School, Pediatric Research Center, Carl-Neuberg-Str. 1, 30625 Hannover, Germany; 3 Center for Chronically Sick Children, Pediatric Endocrinology, University Medicine, Charitè Berlin, Germany

**Keywords:** Rickets, Osteomalacia, Vitamin D, Fibroblast growth factor 23, X-linked hypophosphatemia, Vitamin D-dependent rickets, Nutritional rickets

## Abstract

Rickets is a disease of the growing child arising from alterations in calcium and phosphate homeostasis resulting in impaired apoptosis of hypertrophic chondrocytes in the growth plate. Its symptoms depend on the patients’ age, duration of disease, and underlying disorder. Common features include thickened wrists and ankles due to widened metaphyses, growth failure, bone pain, muscle weakness, waddling gait, and leg bowing. Affected infants often show delayed closure of the fontanelles, frontal bossing, and craniotabes. The diagnosis of rickets is based on the presence of these typical clinical symptoms and radiological findings on X-rays of the wrist or knee, showing metaphyseal fraying and widening of growth plates, in conjunction with elevated serum levels of alkaline phosphatase. Nutritional rickets due to vitamin D deficiency and/or dietary calcium deficiency is the most common cause of rickets. Currently, more than 20 acquired or hereditary causes of rickets are known. The latter are due to mutations in genes involved in vitamin D metabolism or action, renal phosphate reabsorption, or synthesis, or degradation of the phosphaturic hormone fibroblast growth factor 23 (FGF23). There is a substantial overlap in the clinical features between the various entities, requiring a thorough workup using biochemical analyses and, if necessary, genetic tests. Part I of this review focuses on the etiology, pathophysiology and clinical findings of rickets followed by the presentation of a diagnostic approach for correct diagnosis. Part II focuses on the management of rickets, including new therapeutic approaches based on recent clinical practice guidelines.

## Introduction

**Fig. 1:**
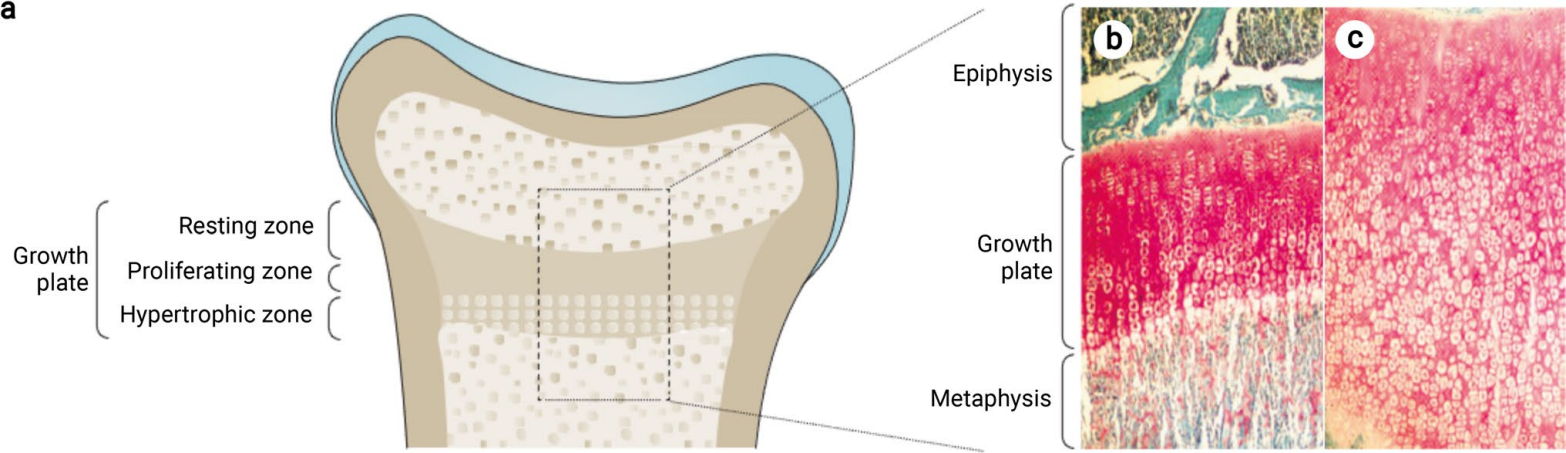
Morphology of the growth plate in rickets. (a,b) Morphology of a healthy, human growth plate (physis). The growth plate is characterized by maturation of chondrocytes (cartilage cells) occurring progressively from the epiphysis to the metaphysis. The border between the metaphysis and the growth plate is marked by a provisional zone of calcification of the cartilage matrix (red–pink staining) which undergoes resorption and replacement with mineralized bone (turquoise staining). **(c)** A rachitic growth plate showing a marked increase in longitudinal width, marked by the persistence of the zone of hypertrophic chondrocytes with lost columnar arrangement. The growth plate abnormalities are the consequence of impaired chondrocyte apoptosis and impaired mineralization of the cartilage matrix surrounding the apoptotic chondrocytes. Apoptosis of hypertrophic chondrocytes is induced by extracellular phosphate via phosphorylation of mitogen-activated protein kinase (MAPK) pathway intermediates and downstream inhibition of the caspase-9-dependent mitochondrial apoptotic pathway. Thus, reduction in ambient phosphate availability to the chondrocyte, which is common to all forms of rickets, seems to be central to the impaired apoptosis. The ligand 1,25-dihydroxyvitamin D and its receptor may also be involved. Finally, expansion of the hypertrophic chondrocyte zone can be induced by impaired vascularization, influenced by vascular endothelial growth factor, which is regulated by MAPK pathway intermediates. Figure reproduced from Carpenter et al. with permission [1]

Rickets is a heterogeneous group of acquired and inherited diseases resulting in disturbances in calcium and/or phosphate heomeostasis and, thereby, affecting the growing skeleton. Rickets is characterized by impaired apoptosis of hypertrophic chondrocytes, resulting in widening of the growth plates in bones and is usually associated with osteomalacia (Fig. 1) [1]. The latter is characterized by an inadequate mineralization of the organic component of the bone matrix—the osteoid—by calcium salts, resulting in soft bone. Historically, rickets was classified as calcipenic or phosphopenic rickets. The latter is also called hypophosphatemic rickets. However, there is growing evidence that the ultimate cause of rickets is an insufficient availability of phosphate required for terminal differentiation and mineralization of growth plate chondrocytes [1–4].

**Fig. 2:**
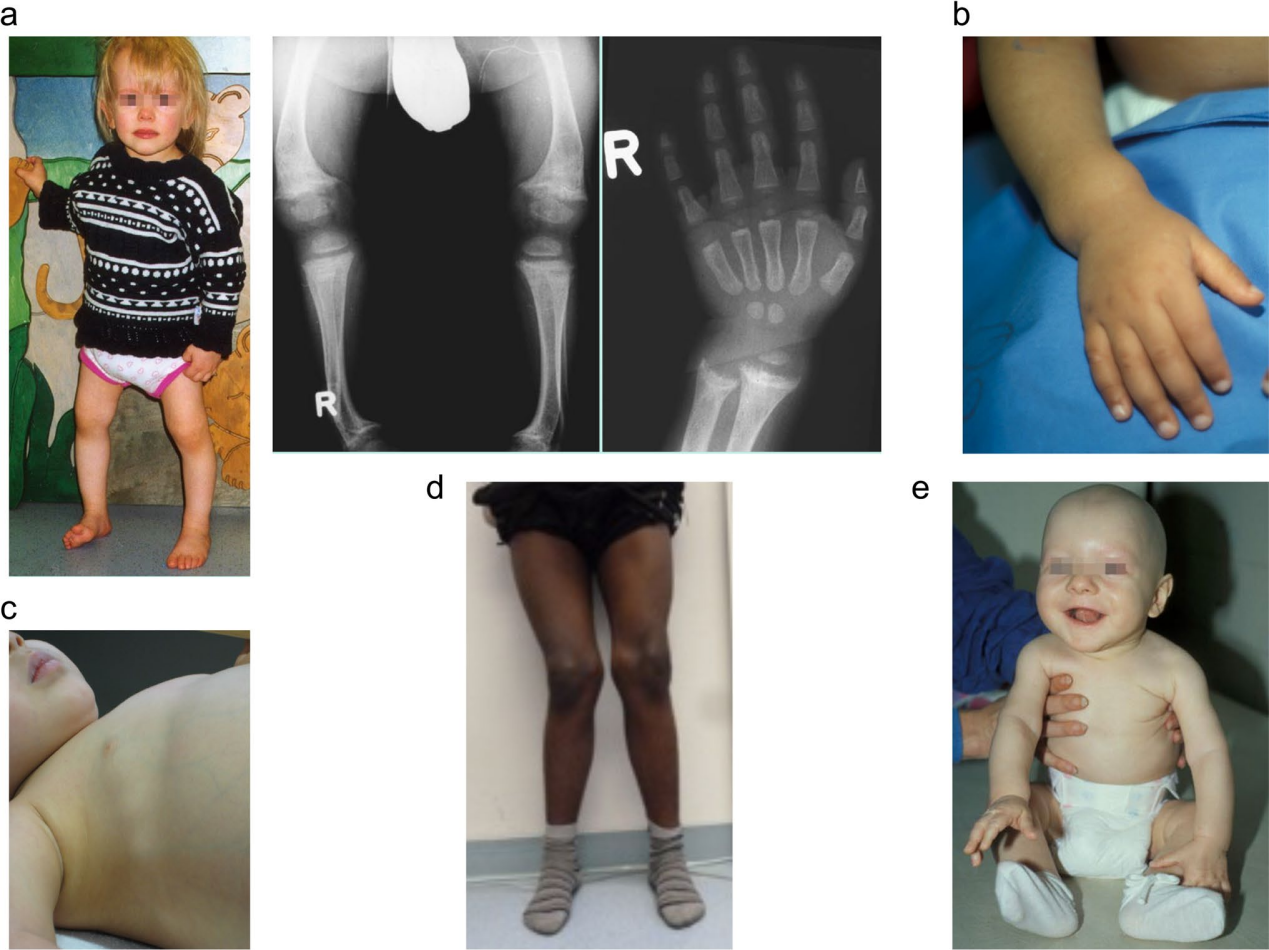
Clinical features of calcipenic rickets. (a) 18-month-old girl presenting with genu vara, and widening of the growth plates and metaphyseal fraying on X-rays, caused by nutritional rickets. (**b, c**) Two infants with widening of the wrist and rachitic rosary, respectively, due to nutritional rickets. (**d**) 14-year-old boy with genu valga due to nutritional rickets. (**e**) Infant with alopecia due to vitamin D-dependent rickets type 2A. Figure 2a, b, and e are reproduced from Schnabel and Haffner with permission [109]

**Fig. 3:**
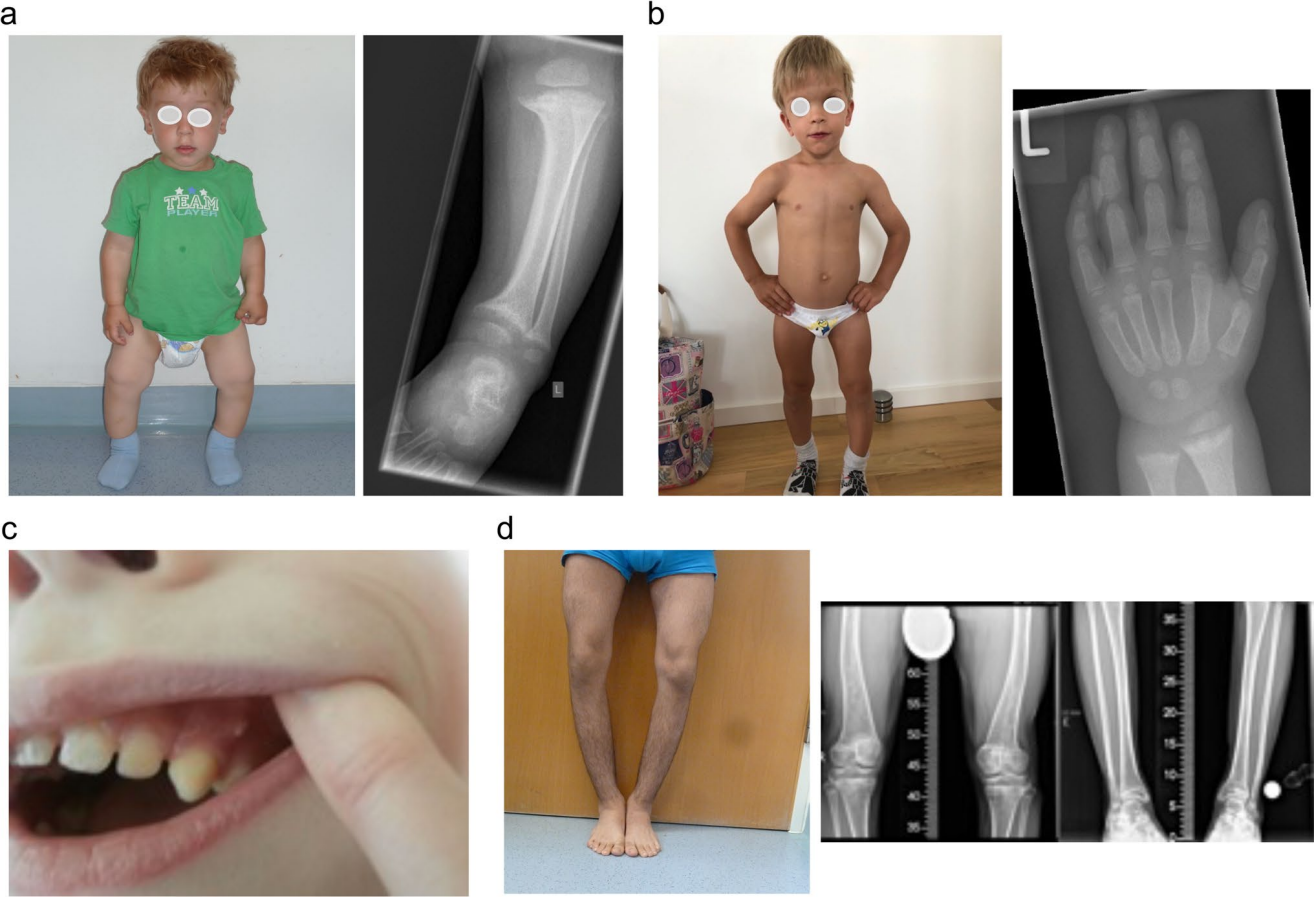
Clinical features of phosphopenic rickets. (a) 2-year-old boy diagnosed with X-linked hypophosphatemia (XLH) at the age of 2 years, presenting with disproportionate short stature (–2.3 SD score), genu vara, and widening of growth plates and metaphyseal fraying on X-rays**. (b)** 3-year-old patient with XLH started on treatment with active vitamin D and phosphate at the age of 2 years showing disproportionate short stature (height, –2.4 SD score), frontal bossing, dolichocephalus and mild signs of rickets on X-ray. **(c)** Dental abscess on an apparently healthy tooth in a child with XLH. **(d)** 16-year-old boy with autosomal-recessive hypophosphatemic rickets type 2 (ARHR2) showing genu vara and mild ricketic signs on X-ray. Figure 3c reproduced with permission from Haffner and Linglart [110]

Clinical presentation depends on age at onset, duration of disease, and underlying pathophysiology. Common features are thickened wrists and ankles due to widened metaphyses, growth failure, bone pain, muscle weakness, waddling gait, and leg bowing. Severe rickets in infancy may also include delayed closure of the fontanelles, parietal and frontal bossing and craniotabes (soft skull bones). Additional features may be present depending on the underlying disease, e.g., symptomatic hypocalcemia (seizures or tetany) in nutritional rickets—the most common cause of calcipenic rickets—and craniosynostosis, dental abscesses, and hearing loss in X-linked hypophosphatemia (XLH)—the most common cause of phosphopenic rickets. Impaired mineralization of the growth plate can be confirmed by typical radiography of the wrist or knee, showing metaphyseal fraying and widening of growth plates, in conjunction with elevated serum levels of the osteoblast marker alkaline phosphatase (ALP) (Figs. 2 and 3) [5, 6].

The discovery of new calcium and phosphate metabolism regulators, such as the phosphaturic hormone fibroblast growth factor 23 (FGF23), together with the elucidation of the underlying genetic defects in many hereditary forms of rickets and the availability of comprehensive genetic testing has improved our understanding of the underlying pathophysiology and revolutionized its diagnosis. Currently, more than 20 inherited and acquired causes of rickets are known, which show a considerable overlap in their clinical findings (Table 1) [1, 7]. Wrong or delayed diagnosis may result in inappropriate treatment of rickets and poor patient outcome. The diagnostic approach to rickets is primarily based on medical history, biochemical tests and radiography, which is followed, if necessary, by genetic tests. The latter is of especial importance in patients suspected to have a genetic form of rickets but no family history of the disease. In addition, targeted therapy has become available for some hereditary forms of rickets, such as XLH which requires correct diagnosis before treatment [8].

**Table 1:** Molecular, genetic and biochemical characteristics of inherited or acquired causes of phosphopenic rickets in comparison to calcipenic rickets. Adapted from Haffner et al. [7]

Disorder (abbreviation; OMIM#)	Gene (location)	Ca	Pi	ALP	U_Ca/Crea_	U_P/Crea_	TmP/GFR	FGF23	PTH	25 (OH)D^a^	1,25 (OH)_2_D	Pathogenesis
**Rickets and/or osteomalacia with high PTH levels (calcipenic rickets)**												
Nutritional rickets (vitamin D and/or calcium deficiency)	NA	N, ↓	N, ↓	↑↑↑	↓	Varies	↓	N	↑↑↑	↓↓, N	varies	Vitamin D deficiency
Vitamin D-dependent rickets type 1A (VDDR1A; OMIM#264700)	*CYP27B1* (12q14.1)	↓	N, ↓	↑↑↑	↓	Varies	↓	N, ↓	↑↑↑	N	↓	Impaired synthesis of 1,25 (OH)_2_D
Vitamin D-dependent rickets type 1B (VDDR1B; OMIM#600081)	*CYP2R1* (11p15.2)	↓	N, ↓	↑↑↑	↓	Varies	↓	N	↑↑↑	↓↓	varies	Impaired synthesis of 25 (OH)D
Vitamin D-dependent rickets type 2A (VDDR2A; OMIM#277440)	*VDR* (12q13.11)	↓	N, ↓	↑↑↑	↓	Varies	↓	N, ↓	↑↑↑	N	↑↑	Impaired signaling of the VDR
Vitamin D-dependent rickets type 2B (VDDR2B; OMIM#264700)	*HNRNPC*	↓	N, ↓	↑↑↑	↓	Varies	↓	N	↑↑↑	N	↑↑	Impaired signaling of the VDR
Vitamin D-dependent rickets type 3 (VDDR3; OMIM# pending)	*CYP3A4*	↓	↓	↑↑↑	↓	Varies	↓	?	↑↑↑	↓	↓	↑ inactivation of 1,25 (OH)_2_D
**Phosphopenic rickets**												
Rickets and/or osteomalacia due to dietary phosphate deficiency or impaired bioavailability												
Breastfed very low birthweight infants Use of elemental or hypoallergenic formula diet or parental nutrition Excessive use of phosphate binders Gastrointestinal surgery or disorders	NA	N, ↑	↓	↑, ↑↑	?	↓	N^b^	N, ↓	N	N	N, ↑	Phosphate deficiency
Rickets and/or osteomalacia with renal tubular phosphate wasting due to elevated FGF23 levels and/or signaling												
X-linked hypophosphatemia (XLH; OMIM#307800)	*PHEX* (Xp22.1)	N	↓	↑, ↑↑	↓	↑	↓	↑, N	N, ↑^c^	N	N^d^	↑ FGF23 expression in bone and impaired FGF23 cleavage
Autosomal dominant hypophosphatemic rickets (ADHR; OMIM#193100)	*FGF23* (12p13.3)	N	↓	↑, ↑↑	↓	↑	↓	↑, N	N, ↑^c^	N	N^d^	FGF23 protein resistant to degradation
Autosomal recessive hypophosphatemic rickets 1 (ARHR1; OMIM#241520)	*DMP1* (4q22.1)	N	↓	↑, ↑↑	↓	↑	↓	↑, N	N, ↑^c^	N	N^d^	↑ FGF23 expression in bone
Autosomal recessive hypophosphatemic rickets 2 (ARHR2; OMIM#613312)	*ENPP1* (6q23.2)	N	↓	↑, ↑↑	↓	↑	↓	↑, N	N, ↑^c^	N	N^d^	↑ FGF23 expression in bone
Raine syndrome-associated (ARHR3; OMIM#259,775)	*FAM20C* (7q22.3)	N	↓	↑, ↑↑	?	↑	↓	↑, N	N, ↑^c^	N	N^d^	↑ FGF23 expression in bone
Fibrous dysplasia (FD; OMIM#174800)	*GNAS (*20q13.3)	N, ↓	↓	↑, ↑↑	↓	↑	↓	N, ↑	N, ↑^c^	N	N^d^	↑ FGF23 expression in bone
Tumor-induced osteomalacia (TIO)	NA	N, ↓	↓	↑, ↑↑	↓	↑	↓	N, ↑	N, ↑^c^	N	N^d^	↑ FGF23 expression in tumoral cells
Cutaneous skeletal hypophosphatemia syndrome (SFM; OMIM#163200)	*NRAS (1p13.2)* *HRAS (11p15.5)* *KRAS (12p12.1)*	N, ↓	↓	↑, ↑↑	↓	↑	↓	N, ↑	N, ↑^c^	N	N^d^	↑ FGF23 expression in dysplastic bone lesions
Osteoglophonic dysplasia (OGD) (OMIM#166250)	*FGFR1* (8p11.23)	N	↓	↑, N	N	↑	↓	N	N, ↑^c^	N	N^d^	↑ FGF23 expression in bone
Hypophosphatemic rickets and hyperparathyroidism (OMIM#612089)	*KLOTHO* (13q13.1)	N	↓	↑, ↑↑	↓	↑	↓	↑	↑↑	N	N^d^	Unknown; translocation of the *KLOTHO* promoter
Rickets and/or osteomalacia due to primary renal tubular phosphate wasting												
Hereditary hypophosphatemic rickets with hypercalciuria (HHRH; OMIM#241530)	*SLC34A3* (9q34.3)	N	↓	↑(↑↑)	N, ↑	↑	↓	↓	Low N, ↓	N	↑↑	Loss of function of NaPi2c in the proximal tubule
X-linked recessive hypophosphatemic rickets (Dent disease 1; OMIM#300554)	*CLCN5* (Xp11.23)	N	↓	↑(↑↑)	N, ↑	↑	↓	varies	varies	N	↑	Loss of function of CLCN5 in the proximal tubule
Hypophosphatemia and nephrocalcinosis (NPHLOP1; OMIM#612286) Fanconi reno-tubular syndrome 2 (FRTS2; OMIM#613388)	*SLC34A1* (5q35.3)	N	↓	↑(↑↑)	↑	↑	↓	↓	varies	N	↑	Loss of function of NaPi2a in the proximal tubule
Cystinosis (OMIM#219800) and other hereditary forms of Fanconi syndrome	*CTNS* (17p13.2)	N, ↓	↓	↑(↑↑)	N, ↑	↑	N, ↓	N, ↑^e^	N, ↑^e^	N	N^d^	Cystine accumulation in the proximal tubule
Iatrogenic proximal tubulopathy	NA	N	↓	↑(↑↑)	varies	↑	↓	↓	varies	N	↑	Drug toxicity

N = normal; ↑ = elevated; ↑↑ or ↑↑↑ = very elevated; ↑ (↑↑) = may range widely: Ca, serum levels of calcium; Pi, serum levels of phosphate; ALP alkaline phosphatase; U_Ca/crea_, urinary calcium to creatinine ratio; U_P/Crea_, urinary phosphate to creatinine ratio; TmP/GFR = maximum rate of renal tubular reabsorption of phosphate normalized to the glomerular filtration rate; FGF23 = fibroblast growth factor 23; PTH = parathyroid hormone; 1,25(OH)_2_D = 1,25-dihydroxyvitamin; 25(OH)D = calcidiol; NA = not applicable; ^a^ = cave: prevalence of vitamin D deficiency was reported to be up to 50% in healthy children; ^b^ = normal after restoration of Pi, but falsely reduced before restoration; ^c^ = PTH may be moderately elevated; ^d^ = decreased relative to the serum phosphate concentration; ^e^ = depending on the stage of chronic kidney disease

In part I of this review, we give an overview on the etiology, pathophysiology and clinical findings of rickets, followed by a diagnostic approach and discussion of important pitfalls in finding the correct diagnosis. Part II focuses on the treatment of rickets, including new therapeutic approaches based on recent clinical practice guidelines.

## Calcium and phosphate homeostasis

**Fig. 4:**
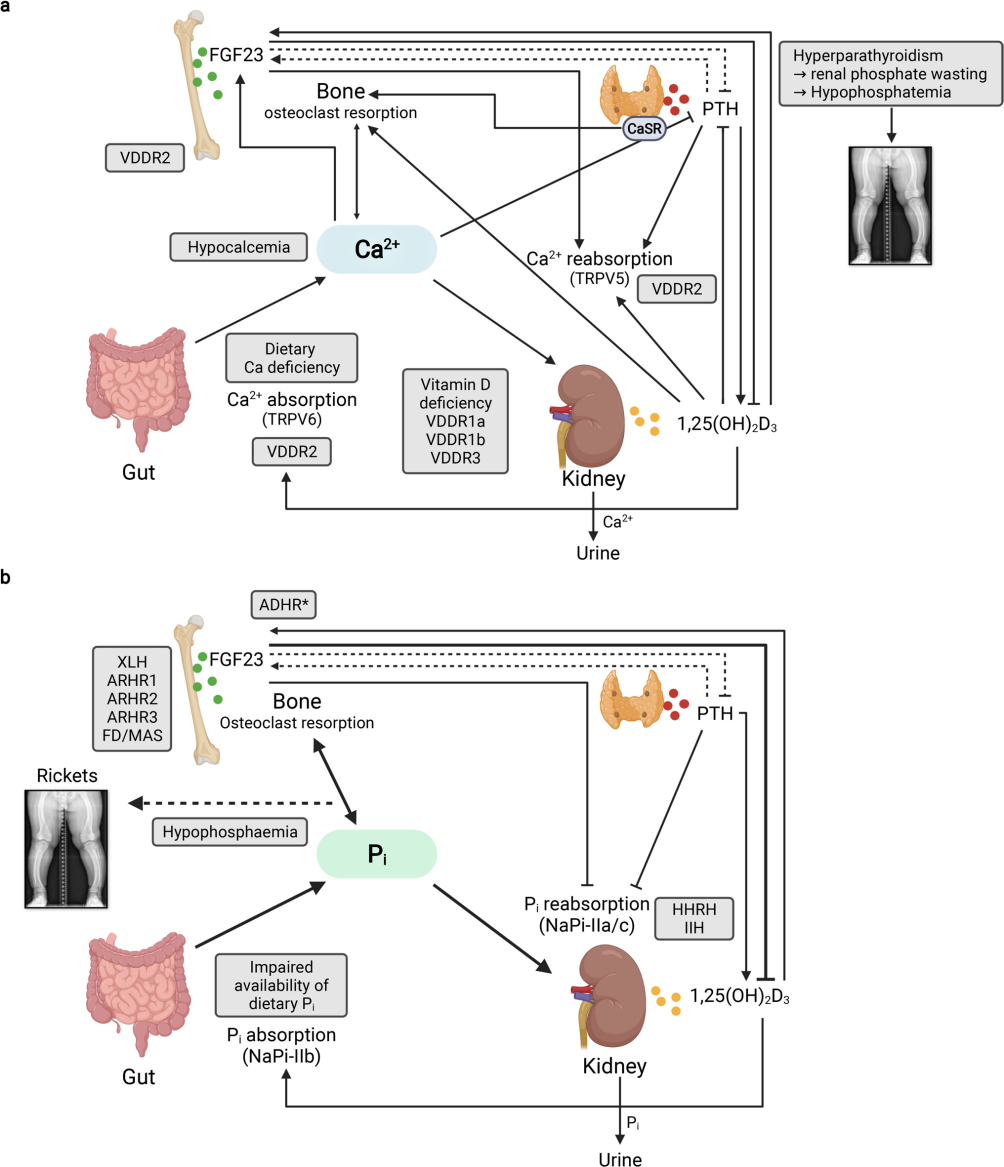
Regulation of calcium (A) phosphate (B) homeostasis. (**A**) The parathyroid gland senses extracellular calcium (Ca^++^) levels and secretes parathyroid hormone (PTH). PTH secretion is stimulated by low Ca^++^ and suppressed by high Ca^++^ plasma concentrations, respectively. PTH stimulates resorption of Ca^++^ from the bone, as well as renal Ca^++^ reabsorption. PTH also stimulates renal 1,25(OH)2D synthesis, and thereby enhances osteoclastic resorption of Ca^++^ from bone, as well as renal calcium reabsorption via TRPV5 and suppresses PTH synthesis. Circulating fibroblast growth factor 23 (FGF23) originates mainly from osteocytes. FGF23 suppresses both renal 1,25(OH)2D production and PTH. Both Ca^++^ and 1,25(OH)_2_D stimulate FGF23 production. The sites of defects of the different causes of hypocalcemic disorders (also called calcipenic rickets) are given in the purple boxes. This includes nutritional rickets due to vitamin D deficiency and/or impaired dietary calcium availability and genetic defects in vitamin D metabolism or action (vitamin D-dependent rickets (VDDR types 1–3)). Note: hypophosphatemia due to secondary hyperparathyroidism-associated renal phosphate wasting, rather than hypocalcemia ultimately causes rickets in calcipenic rickets. (**B**) FGF23 and PTH reduce renal tubular phosphate (Pi) reabsorption by reducing the apical expression of the sodium–phosphate cotransporters NaPi IIa and NaPi IIc. PTH stimulates, while FGF23 inhibits 1,25(OH)_2_D production. 1,25(OH)_2_D increases intestinal absorption of dietary Pi by enhancing NaPi IIb expression and stimulates FGF23 synthesis. PTH and FGF23 affect each other’s production through a negative feedback loop by as yet unknown mechanisms. The sites of defects of the different causes of hypophosphatemic disorders (“phosphopenic rickets”) are given in the purple boxes. This includes impaired dietary phosphate availability and genetic defects: XLH, X-linked hypophosphatemia; ARHR1/2/3, autosomal recessive hypophosphatemic rickets 1/2/3; FD/MAS, Fibrous dysplasia/McCune-Albright syndrome; HHRH, hereditary hypophosphatemic rickets with hypercalciuria; IIH, idiopathic hypercalcemia; ADHR, autosomal dominant hypophosphatemic rickets. *FGF23-protein resistant to degradation. Created with BioRender.com

Mineral homeostasis is maintained by complex interactions between organ systems—primarily the skeleton, intestine and the kidney. It is regulated by several hormones, including calciotropic and phosphate-regulating hormones, e.g., parathyroid hormone (PTH), vitamin D metabolites and FGF23 (Fig. 4). Adequate intake of calcium and phosphate is mandatory in order to maintain bone integrity and allow normal skeletal growth in children. Most of the calcium (> 99%) is stored in the skeleton, and less than 0.5% circulates in the blood, with approximately 50% being bound to albumin or globulin. The primary sources of dietary calcium in children are milk products. Typically, about 35% of dietary calcium is absorbed in the small intestine via transepithelial transport through the apical membrane calcium channel TRPV6, active extrusion across the basolateral membrane by PMCA1b and/or other mechanisms. Thereafter, calcium enters the extracellular fluid, and is deposited in bone, secreted back to the gut, or filtered and later partly reabsorbed via the TRPV5 channel within the kidneys. Children have a positive net balance of calcium which peaks in infancy and puberty, the periods of highest growth rates. Adequate availability of calcium is essential for hydroxyl-apatite [Ca_5_(PO_4_)_3_OH] formation in bone. Influx of ionized calcium (Ca^++^) regulates many biological processes, including neural transmission, muscle contraction, and protein secretion. Circulating calcium represents a central mediator between bone, parathyroid glands, the intestine and the kidneys and is kept constant, within close limits, by numerous hormonal signals.

Inorganic phosphate (Pi) is of major importance for the maintenance of cellular metabolism and skeletal mineralization. Pi contributes to approximately 0.6 and 1% of body weight in neonates and adults, respectively. The skeleton and teeth contain the vast majority (85%) of total body Pi in the form of hydroxyl-apatite. Approximately 14% is distributed within the intracellular compartment. Inorganic phosphate plays an important role in many cellular processes, e.g., energy metabolism (ATP), cell membrane function (phospholipids), cell signaling (phosphorylation by kinases), and DNA- or RNA-biosynthesis (phosphorylated nucleotides) [9]. The extracellular compartment contains only 1% of the total body Pi content, where it serves as an acid–base buffer in plasma and urine. As phosphate measurements in clinical practice are limited to serum and urine, only “the tip of the iceberg” is visible. Similarly to serum calcium, circulating Pi represents a central mediator between bone, parathyroid glands, the intestine and the kidneys, and is kept constant, within close limits, similarly to calcium by numerous hormonal signals (Fig. 4B).

After completion of skeletal growth, a steady state can be assumed where serum Pi concentration is kept constant by a balanced equilibrium between dietary phosphate absorption in the intestine (16 mg/kg per day), bone-turnover of phosphate (3 mg/kg per day) and urinary phosphate excretion (16 mg/kg per day), (Fig. 4B). By contrast, a positive Pi balance is required in children and adolescents to allow for normal skeletal growth. Therefore, adequate alimentary Pi is important and can be provided by protein-rich foods such as meat, milk and eggs. However, in contrast to calcium, phosphate is usually abundant in the Western diet, and a dietary phosphate deficiency is rarely observed. After intestinal absorption, Pi distributes in the extracellular compartment and equilibrates with the bone and intracellular compartment. Within the kidney, Pi is freely filtered across the capillaries of the glomerulus and then reabsorbed, mainly by the proximal renal tubules, depending on the actual needs of the body. The type II family of sodium-coupled phosphate-transporters, i.e., NaPi2a (encoded by *SLC34A1*), NaP2b (encoded by *SLC34A2*), and NaPi2c (encoded by *SLC34A3*) ensures the bulk of the intestinal and renal transepithelial Pi transport [10–12]. Approximately 90% of phosphate reabsorption within the kidney occurs via NaPi2a, which is localized at the brush border of renal proximal tubular cells. The rest is handled by NaPi2c within the proximal tubules of deep nephrons. Genetic defects of NaPi2A and NaPi2C result in renal phosphate wasting, causing hypophosphatemia and nephrocalcinosis, also known as idiopathic infantile hypercalcemia (IIH) and hypophosphatemic rickets with hypercalciuria (HHRH), respectively [13–15]. Hypercalcemia and hypercalciuria result from an enhanced 1,25(OH)_2_D synthesis. Absorption of dietary phosphate in the small intestine is carried out by NaPi2b which is localized in the brush border membrane of enterocytes and via passive paracellular diffusion [12].

### Calcium and phosphate homeostasis regulators

The amount of intestinal calcium and phosphate absorption mainly depends on dietary calcium and Pi intake. Both are partially regulated by 1,25-dihydroxyvitamin D (1,25(OH)_2_D) [11, 12]. In addition, dietary Pi intake directly affects the amount of renal phosphate reabsorption. Renal phosphate handling is also regulated via phosphaturic hormones – parathyroid hormone (PTH) and FGF23 – both affecting the expression of NaPi2a and NaPi2c within the proximal tubular brush border membranes, in order to keep serum Pi within the normal range (Fig. 4B). Insulin-like growth factor 1 (IGF-1)—the main mediator of growth hormone—increases Pi reabsorption in the renal proximal tubules via upregulation of NaPi2a and NaPi2c, resulting in a positive Pi balance. Both, PTH and 1,25(OH)_2_D enhance calcium reabsorption in the distal renal tubules by upregulation/activation of the epithelial calcium channel TRPV5.

*Parathyroid hormone* synthesis and secretion is stimulated via the calcium-sensing receptor by low serum Ca^++^ and 1,25(OH)_2_D levels and elevated serum Pi concentrations, and is down-regulated by elevated serum Ca and 1,25(OH)_2_D concentrations and low Pi levels [16]. It inhibits NaPi-cotransport by enhancing the clearance of NaPi2a from the tubular epithelial brush border membrane and directly stimulates renal calcium reabsorption (vide supra). It also stimulates the synthesis of 1,25(OH)_2_D, resulting in increased intestinal Pi and Ca^++^ absorption. The total effect of these actions is an enhancement of circulating Ca^++^ levels and a reduction in circulating Pi levels (Fig. 4).

*Fibroblast Growth Factor 23* is mainly synthesized in bone (osteocytes and osteoblasts) and teeth. Its synthesis is stimulated by Pi, Ca^++^, PTH, and vitamin D [17, 18]. FGF23 synthesis in bone is mainly regulated by two genes—phosphate-regulating neutral endopeptidase homolog (*PHEX*) and dentin matrix acidic phosphoprotein (*DMP1*)—by poorly understood mechanisms [19]. FGF23 requires binding to its co-receptor Klotho, a membrane-bound protein with ß-glucuronidase activity, to activate FGF-receptor 1 (FGFR1). Activation of FGFR1 results in inhibition of proximal tubular Pi reabsorption via triggering internalization and degradation of NaPi2a and NaPi2c, suppresses renal 1,25(OH)_2_D synthesis, and stimulates 1,25(OH)_2_D degradation. The total effect is a decrease in circulating Pi and 1,25(OH)_2_D concentrations (Fig. 4).

**Fig. 5:**
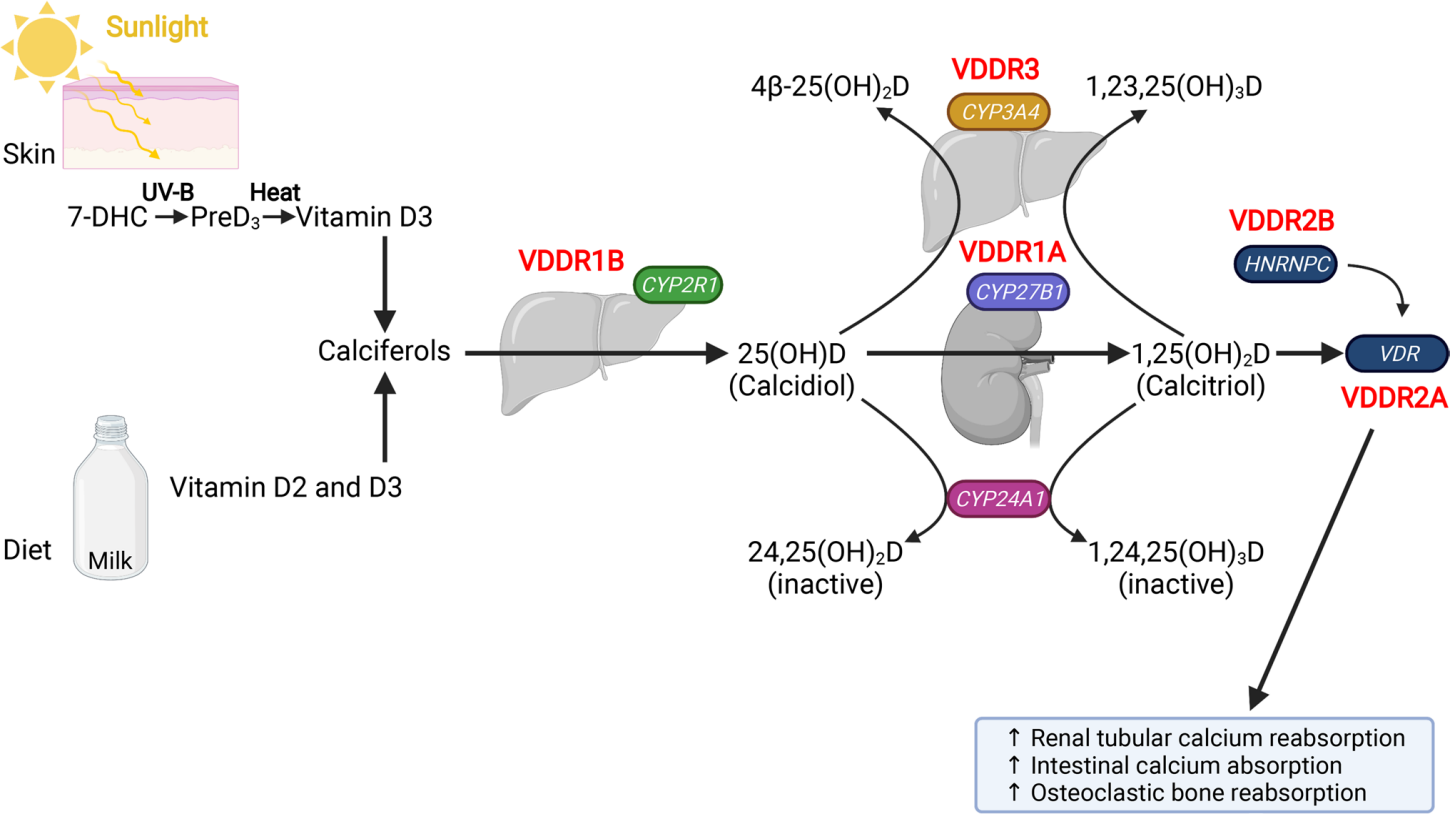
Vitamin D homeostasis and hereditary causes of impaired VDDR function. The overall metabolic control of vitamin D homeostasis is shown. Synthesis of calciferols via sunlight exposure or dietary intake of vitamin D2 and D3 is given on the left-hand side. The genes involved in the different forms of VDRR are indicated in italics in the rounded boxes. Created with BioRender.com

*Vitamin D*_*3*_ (cholecalciferol) is predominantly formed by sunlight (90%) (ultraviolet B in the 290–315 nm range) on the skin (deep layers of the epidermis) from 7-dehydrocholesterol (Fig. 5) [20]. The smaller portion of the daily vitamin D requirement is absorbed enterally (vitamin D_2_ from plant products, vitamin D_3_ from animal products). After binding to a specific protein (DBP), vitamin D_2_ and D_3_ are transported to the liver. In liver microsomes, hydroxylation occurs in 25-hydroxyvitamin D_3_ (25(OH)D (also known as calcidiol) via 25-hydroxylase (encoded by *CYP2R1*). After transport via vitamin D-binding protein (DBP) to the kidneys, a second hydroxylation occurs via 1α-hydroxylase (encoded by *CYP27B1*) to active vitamin D (1,25(OH)_2_D (also known as calcitriol). Whilst decreases in serum calcium and/or phosphate levels stimulate this hydroxylation, hypercalcemia, hyperphosphatemia, and elevated 1,25(OH)_2_D concentrations decrease it, and stimulate the conversion of 25OHD and 1,25(OH)_2_D via cytochrome P450 24A1 (encoded by *CYP24A1*) to biologically less effective 24,25(OH)_2_D and 1,24,25(OH_2_)D in the renal tubules. Vitamin D metabolites can also be inactivated by cytochrome P450 3A4 (encoded by *CYP3A4*) which is highly expressed in the liver.

*Calcitriol* and its precursors execute their genomic effects via the intracellular nuclear receptor termed VDR. Their non-genomic actions are mediated by binding to a presumptive plasma membrane receptor. Thereby, calcitriol can directly stimulate renal tubular calcium reabsorption via upregulation of the expression of the epithelial calcium channel TRPV5 and increase intestinal calcium and phosphate absorption via stimulation of the epithelial calcium channel TRPV6 and phosphate transporter NaPi2b, respectively [21–23]. This results in a positive calcium and phosphate net balance. Furthermore, 1,25(OH)_2_D stimulates osteoblasts to increase cytokine synthesis and, thereby, osteoclastogenesis and bone resorption. Compared to vitamin D_3_, 25(OH)D is metabolically tenfold, and 1,25(OH)_2_D 1,000-fold more potent.

## Pathophysiology of rickets

**Fig. 6:**
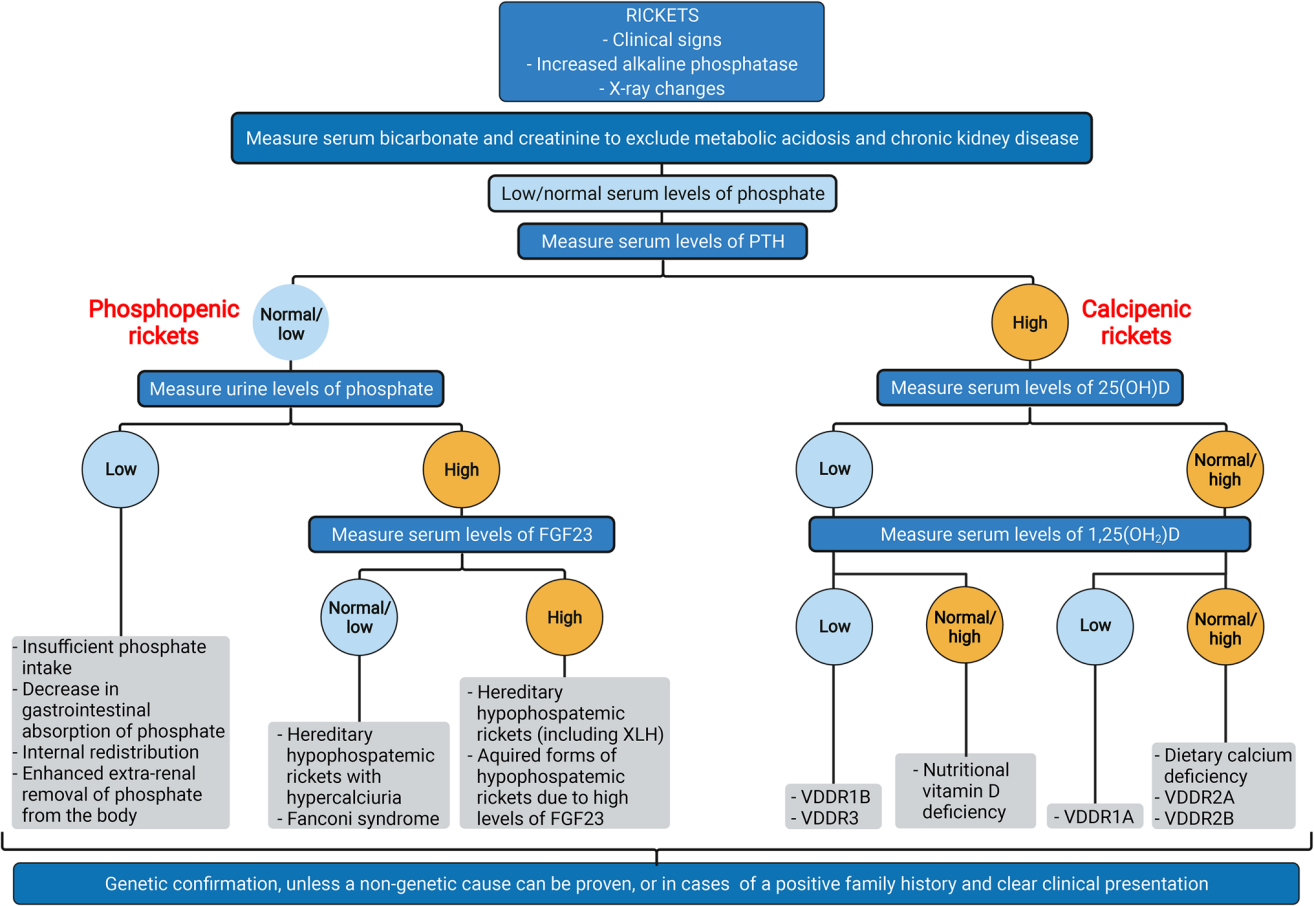
Algorithm for the evaluation of a child presenting with rickets. The differential diagnoses are based on the mechanisms leading to hypophosphatemia, i.e., high parathyroid hormone (PTH) activity (calcipenic rickets), inadequate intestinal phosphate absorption, or renal phosphate wasting (phosphopenic rickets). The latter may be due to either primary tubular defects or high serum FGF23 concentratons. Further details are given in Table 1; FGF23, fibroblast growth factor 23. Created with BioRender.com

It was previously assumed that rickets primarily resulted from disturbances of calcium and vitamin D metabolism and was classified into two major groups, based on the leading biochemical alteration, into calcipenic and phosphopenic rickets. However, recent data provides strong evidence that the real culprit is insufficient availability of phosphate which is a prerequisite for terminal differentiation of growth plate chondrocytes [2–4]. Endochondral bone growth is characterized by an orderly transformation of growth plate chondrocytes into bone [24]. Cartilage cells of the "resting zone", adjacent to the epiphysis, undergo maturation to chondrocytes which are organized in columns aligned along the longitudinal axis and hypertrophy with time. Thereafter, terminally differentiated hypertrophic chondrocytes are replaced via vascular invasion, apoptosis, mineralization and invasion of osteoclasts, resulting in primary cancellous bone. Studies in various animal models of rickets show that reduced concentrations of extracellular phosphate impair caspase-9-mediated apoptosis of hypertrophic chondrocytes, causing the characteristic rachitic changes (Fig. 1) [2, 4]. This led to the concept that hypophosphatemia is the denominator of all forms of rickets. Indeed, both forms of rickets—calcipenic and phosphopenic—are associated with low serum Pi levels. Therefore, the underlying causes of hypophosphatemia are the basis of the diagnostic algorithm given in Fig. 6 which is described below. However, the historical terms calcipenic and phosphopenic rickets will still be used in this review and are integrated in the algorithm for didactic reasons.

### Calcipenic rickets

**Fig. 7:**
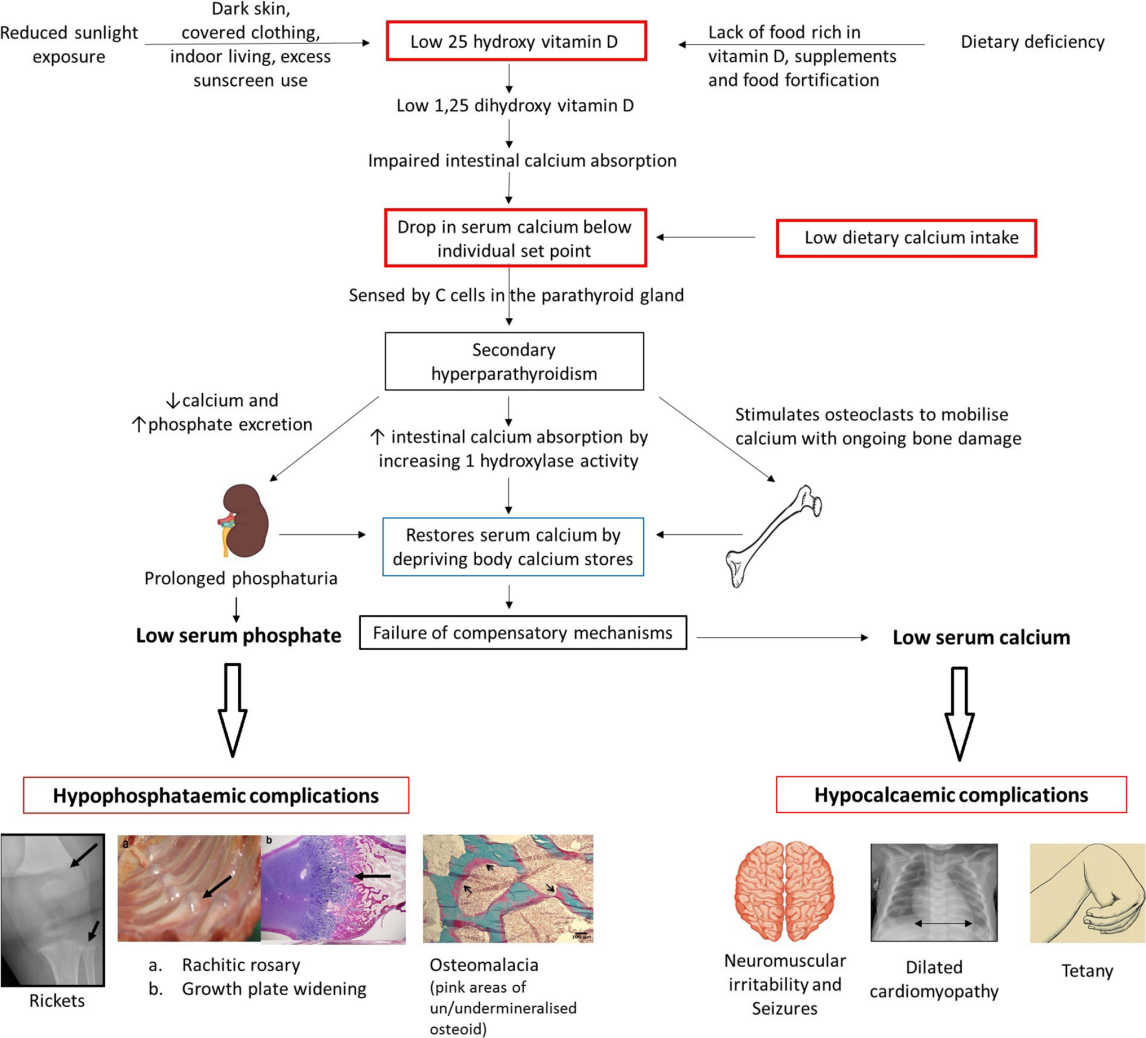
Pathophysiology of nutritional rickets, due to vitamin D deficiency and/or dietary calcium deficiency. Both etiologies result in calcium deprivation and hyperparathyroidism occurs in an attempt to maintain normal serum calcium levels. In the long run, calcium deprivation and phosphate loss result in hypocalcaemic and hypophosphataemic complications. Figure reproduced from Uday and Högler with permission [29]

Calcipenic rickets results from impaired calcium availability, which is either due to reduced calcium intake and/or vitamin D deficiency (nutritional rickets) or impaired action of 1,25(OH)_2_D (vitamin D-dependent rickets) (VDRR)) [1, 25, 26]. In general, the reduced availability of calcium results in a tendency toward lower serum Ca which stimulates PTH synthesis, in order to maintain normal serum Ca^++^ levels by enhancing renal 1,25(OH)_2_D synthesis and, thereby, intestinal calcium absorption. In addition, PTH causes internalization, and subsequent degradation, of NaPi2a and NaPi2C in the proximal tubule, leading to renal phosphate wasting and consecutive hypophosphatemia. The latter results in rickets and/or osteomalacia. When PTH-driven demineralization of the skeleton cannot further compensate calcium deprivation, patients may present with symptomatic hypocalcemia, i.e., seizures and tetany and heart failure due to dilated cardiomyopathy in infants (Fig. 7).

Nutritional rickets, also called “the English disease,” is still the most common cause of rickets globally [1, 27]. Its prevalence declined during the twentieth century, but recent reports indicate that it is still prevalent, even in so-called developed countries, due to changes in lifestyle (decreased sunlight exposure, which is needed for activation of vitamin D) and nutrition (e.g., vegan diet) and lack of or inconsistent vitamin D prophylaxis during the first year of life. Many risk factors for nutritional rickets have been identified (Table 2). Finally, severely ill children may present with malnutrition and vitamin D deficiency secondary to chronic disease [28–30].

**Table 2:** Risk factors for nutritional rickets

Vitamin D deficiency risk	Calcium deficiency risk
**Skin pigmentation**	Restricted dairy diet
Dark skin produces less vitamin D due to excess melanin which reduces UVB penetration	Cultural reasons
	Food allergies
	Lactose intolerance
	Malnutrition
	Malabsorption
**Sun avoidance**	
Whole body clothing	
Excessive sunscreen use	
Indoor living/ institutionalization	
**Lack of sunlight**	
High‑latitude residence	
Environmental pollution	
**States of high physiological demand**	
Infancy	
Pregnancy and lactation	
Old age	

Individuals with more than one risk factor are at highest risk

UVB, ultraviolet B

Table adapted from Uday and Högler [29]

Clinical presentation largely depends on the age of the patient and the severity and duration of the underlying deficiency [28–30]. Symptomatic hypocalcemia peaks in infancy and puberty, due to the high calcium demands during these periods of rapid growth. Young infants may show irritability and poor feeding and sometimes apnea and stridor. In addition, craniotabes (soft skull) and large fontanelle may be present. Radiological signs of rickets may be mild or even lacking, as prolonged periods of elevated PTH levels are required before they manifest in early infancy. Older infants usually present with the typical features of rickets, including failure to thrive, delayed development, hypotonia, enlarged wrists/ankles, rachitic rosary (enlarged costochondral junctions of the rib) and hypocalcemic seizures. In addition, tachycardia, tachypnea, hepatomegaly and edema suggest the presence of heart failure, due to cardiomyopathy, which is associated with increased risk of death. In children, additional features such as abnormal dentition, leg bowing, and fractures may occur. Adolescents may manifest with symptomatic hypocalcemia, bone pain, muscle weakness, waddling gait, leg bowing and fractures. Symptoms may mimic myopathies, often resulting in delayed diagnosis (Table 3).

**Table 3:** Typical clinical features of certain causes of rickets

Clinical features	Suggested underlying disease
*At all ages:* Symptomatic hypocalcemia: seizures, tetany, and hypotonia *Young infants:* irritability, poor feeding, apnea, stridor, craniotabes, large fontanelle	Nutritional rickets and VDDR
*Older infants:* failure to thrive, delayed development, hypotonia, frontal bossing, thickened wrists and ankles (widened metaphysis) and enlarged costochondral junctions of the ribs (rachitic rosary), heart failure (tachycardia, tachypnea, hepatomegaly and edema)	Frontal bossing, swollen joints and rachitic rosary, also seen in other forms of rickets
*Children:* abnormal dentition/ enamel hypoplasia, frontal bossing, thickened wrists and ankles (widened metaphysis), leg bowing, fractures	Leg bowing, fractures and frontal bossing, also seen in other forms of rickets
*Adolescents:* bone pain, muscle weakness, waddling gait, leg bowing and fractures	Also noted in other forms of rickets
Partial or complete alopecia	VDDR type 2A and 2B
*At all ages:* disproportionate short stature *Infants:* craniosynostosis	Hereditary forms of FGF23-mediated hypophosphatemia, e.g., XLH, ADHR, ARHR1 and 2
*Children and adolescents:* dental abscesses, hearing loss, thickened wrists and ankles (widened metaphysis), leg bowing, waddling gait, frontal bossing	Enlarged joints, leg bowing, and waddling gait, and frontal bossing also seen in other forms of rickets
*Adolescents:* periodontitis, pseudofractures	
Syringomyelia, Arnold-Chiari malformation,enthesopathy, osteoarthritis (adults)	XLH
Clinical symptoms after early childhood	ADHR, TIO, nutritional rickets
Anemia	ADHR
Café-au-lait macules	McCune Albright syndrome / fibrous dysplasia
Facial dysmorphism, failure of tooth eruption, short stature	Osteoglophonic dysplasia
Craniofacial anomalies including hypoplastic nose, midface hypoplasia, exophthalmus, intracranial calcification, sensorineural hearing loss, developmental delay, epilepsy, large fontanelle, and amelogenesis imperfecta	Raine syndrome
Hypercalciuria, nephrocalcinosis or nephrolithiasis	HHRH, nephropathic cystinosis, Dent disease, distal renal tubular acidosis (dRTA)
Polyuria, polydipsia, fever episodes due to dehydration	Fanconi syndrome, e.g., nephropathic cystinosis

Vitamin D-dependent rickets (VDDR) is a group of rare, inherited defects of vitamin D metabolism (failure to synthesize 25(OH)D, or 1,25(OH)_2_D) or impaired vitamin D receptor signaling), resulting in end-organ resistance to 1,25(OH)_2_D (Fig. 5). To date, 5 types of VDDR have been described [25, 31, 32]. VDDR shares many clinical and biochemical features with nutritional rickets (Tables 1 and 3). Patients with VDDR1A usually become symptomatic in early infancy, presenting with typical skeletal signs of rickets, failure to thrive, hypotonia, irritability, tetany or seizures, and, in cases of late diagnosis, fractures [33]. Patients with VDDR1B show a similar phenotype which may sometimes be much milder and often improves with age [34]. Patients with VDDR2A/B develop severe hypocalcemia in infancy. About half of VDDR2A, but less so with VDDR2B patients develop alopecia, failure to grow eyelashes and eyebrows due to disrupted VDR, as a functional VDR is required for normal hair follicle cycling (Fig. 2e) [35]. VDDR3 is extremely rare and associated with features similar to VDDR1 [31].

### Phosphopenic rickets

Phosphopenic rickets, also called hypophosphatemic rickets, can either be due to dietary phosphate deficiency or impaired bioavailability, FGF23-mediated renal phosphate wasting or to primary or acquired renal tubular phosphate wasting (Fig. 4B, Table 1) [3, 7, 36]. X-linked hypophosphatemia is the most frequent cause of hypophosphatemic rickets and responsible for approximately 80% of all cases. There is considerable overlap between the clinical features of XLH and other forms of hypophosphatemic rickets.

*Dietary phosphate deficiency or impaired availability* may cause rickets, especially in high-risk populations. Phosphate-deficient diets are rare, and nutritional rickets is usually mainly due to impaired calcium intake or vitamin D deficiency [1]. However, very-low-birthweight infants, especially when they are breast-fed and no phosphate supplements are given, may present with rickets due to their high phosphate requirements [37]. In addition, a special elemental or hypoallergenic formula diet may be associated with impaired phosphate bioavailability, under certain clinical circumstances [38, 39]. Gastrointestinal surgery or short bowel syndrome may also result in low phosphate uptake. Finally, excessive use of phosphate binders or too much restriction of phosphate intake, especially in formula-fed infants suffering from chronic kidney disease, must be considered.

*FGF23-mediated renal phosphate wasting* is a group of disorders characterized by increased synthesis of FGF23 or reduced degradation of intact FGF23. *X-linked hypophosphatemia* is a dominant inherited disorder with a prevalence of approximately 4–5/100,000 children and caused by mutations in *PHEX*, underlying in about 90% of familial cases and 70% of sporadic cases of hypophosphatemic rickets [40, 41]. The mechanism by which pathogenic variants in the *PHEX* gene lead to increased circulating FGF23 concentrations is still largely unknown [19]. *PHEX* encodes a membrane-bound endopeptidase and is primarily expressed in bone (osteoblasts and osteocytes), and teeth (odontoblasts) [42] and is thought to affect the expression of FGF23 rather than its degradation [19, 43]. PHEX may regulate serum FGF23 by indirect cleavage of proprotein convertases such as subtilisin/kexin-type 2 (PC2). In addition, *PHEX* malfunction results in increased skeletal synthesis of osteopontin and acid serine aspartate-rich-MEPE-associated protein (ASARM) peptide, both of which also contribute to impaired bone mineralization in XLH [19, 44]. Thus, XLH is due to a complex osteoblast/odontoblast defect which may explain its special clinical features.

Clinical symptoms of XLH usually develop around the age of walking and resemble those of other forms of rickets including growth failure, thickened wrists and ankles due to widened metaphyses, bone deformities, a waddling gait and a large forehead. Characteristically, additional features are disproportionate short stature (short legs, and preserved trunk length), dental abscesses, craniosynostosis, leading to a dolichocephalic shape of the head, and sensorineural hearing loss [7, 45–47]. However, the latter features may also be noted in other forms of hereditary FGF23-mediated hypophosphatemia (vide infra). Tooth eruption is often delayed. Mineralization of dentine is markedly impaired resulting in spontaneous endodontic abscesses and early decay of lacteal and permanent teeth [48]. Type 1 Chiari malformation and syringomyelia are rare, mostly asymptomatic complications of XLH, but may result in headaches and neck pain [45]. Diagnosis may be delayed until adulthood, especially in patients with a negative family history and mild clinical phenotype. Adults with XLH often develop periodontitis, alveolar bone and tooth loss, enthesopathies, (ossification of the entheses, e.g., at the Achilles tendon), osteoarthritis (e.g., of the hip), and pseudofractures (characterized by cortical infraction surrounded by a thickened periosteum on X-ray) which may result in pain, reduced quality of life, and/or immobility [7, 49].

*Autosomal-dominant hypophosphatemic rickets* (ADHR) is a rare disorder, due to activating pathogenic variants of the *FGF23* gene. The resulting mutated protein is resistant to proteolytic cleavage, leading to enhanced FGF23 serum concentrations and, thereby, clinical consequences as in other forms of FGF23 excess [50]. Due to its incomplete penetrance, ADHR patients show a highly variable phenotype and patients usually become symptomatic after childhood [51]. Leading complications due to osteomalacia in adult patients include weakness, fatigue, bone pain and pseudofractures and tumor-induced osteomalacia (TIO) is the most important differential diagnosis (vide infra). Iron status is an important regulator of FGF23 metabolic pathways, and iron deficiency predisposes ADHR patients to become clinically symptomatic [52].

*Autosomal-recessive forms of hypophosphatemic rickets* (ARHR1 and 2) are either caused by pathogenic variants in the dentine matrix acidic phosphoprotein 1 (*DMP1*) gene or in the ectonucleotide pyrophosphatase/phosphodiesterase 1 (*ENPP1*) gene, which are both expressed in bone and teeth [53–56]. The mechanism of how *ENPP1* and *DMP1* gene mutations result in elevated FGF23 serum levels is largely unclear [19]. Clinical symptoms resemble those seen in XLH. DMP1 protein plays an important role in the development of bone, cartilage and teeth, whereas ENPP1 is a regulator of bone mineralization and tissue calcification via formation of inorganic pyrophosphate (PPi). *ENPP1* mutations result in an unbalanced ratio of phosphate and PPi and increased mineral accumulation in tissue and bone, and impaired bone development. Lorenz-Depiereux et al. were the first to describe inactivating mutations in the *ENPP1* gene in 4 families with hypophosphataemic rickets [55]. In ARHR2, in contrast to ENPP1-related GACI, a significantly stronger hypophosphatemia is found, which possibly represents a “protective mechanism” against arterial calcification. *ENPP1* mutations were initially described in cases of infantile arterial calcification [57]. There is a high phenotypic heterogeneity and clinical presentation may differ widely in family members showing the same biallelic *ENPP1* mutations, including enthesopathy and primary hyperparathyroidism [58].

*Raine syndrome* is a very rare disease caused by mutations in *FAM20C,* a major regulator of osteoblasts and osteocytes and stimulator of FGF23 cleavage [59]. Patients present generalized osteosclerosis, and a wide spectrum of other features, including craniofacial anomalies (hypoplasia of the nose/midface), seizures, sensorineural hearing loss, delayed motor development, large fontanelle and abnormal enamel formation. However, the dysmorphic features may be subtle in some patients.

*Tumor-induced osteomalacia* (TIO) is due to overproduction of FGF23 and other phosphaturic factors by benign slow-growing mesenchymal tumors [60]. TIO has rarely been described in children but is an important differential diagnosis in adults presenting with bone pain, muscle weakness, fatigue and/or pseudofractures in conjunction with hypophosphatemia and high levels of circulating FGF23.

*Fibrous dysplasia (FD*) is due to activating mutations in *GNAS* observed in McCune-Albright syndrome (MAS), which is characterized by FD of bone, café-au-lait spots, and precocious puberty, resulting in increased FGF23-synthesis in bone [61]. Hypophosphatemia is usually mild and associated with phosphate-wasting, but may result in bone fractures and bone pain.

Finally, cutaneous disorders such as *epidermal nevi (EN), congenital melanocytic nevi* (CMN) and Schimmelpenning–Feuerstein–Mims syndrome are rare causes of FGF23-excess. Patients show multiple segmental skin and bone lesions, due to activating somatic mutations of *HRAS* or *NRAS,* which are also responsible for the enhanced FGF23-synthesis in bone [62].

*Hypophosphatemic disorders with normal or suppressed FGF23-activity* include *HHRH*, which is due to mutations in *SLC34A3* encoding for NaPi2C [14, 63, 64]. HHRH usually shows increased 1,25(OH)_2_D serum concentrations which promote hypercalciuria, low PTH serum levels, nephrocalcinosis and/or nephrolithiasis. However, clinical symptoms and changes in biochemical parameters may be subtle. The clue is to thoroughly assess the urinary calcium–creatinine ratio in all patients with hypophosphatemic rickets, which is usually normal in XLH but elevated in *HHRH*.

*Hypophosphatemia and nephrocalcinosis* is due to mutations in the *SLC34A1* gene encoding for NaPi2a. Clinical presentations vary widely including idiopathic infantile hypercalcemia (IIH), nephrolithiasis, and rickets associated with renal phosphate wasting [14, 65].

*Osteoglophonic dysplasia* is due to activating mutations in *FGFR1* (Fibroblast growth factor receptor 1) [66, 67]. Clinical symptoms include craniosynostosis, facial dysmorphism, delayed eruption of teeth and growth impairment, which may be associated with hypophosphatemic rickets due to enhanced bone FGF23-synthesis.

Renal Fanconi syndrome is another important cause of renal phosphate wasting, due to a generalized proximal tubular dysfunction [68]. Clinical consequences result mainly from polyuria, hypophosphatemia and metabolic acidosis, i.e., malnutrition, rickets and growth failure. Nephropathic cystinosis represents its most common hereditary form [69]. Incomplete Fanconi syndrome is noted in Dent disease 1, which is due to inactivating mutations in the *CLCN5* gene encoding for CIC-5 expressed in proximal renal tubules, resulting in low molecular weight proteinuria associated with hypercalciuria, nephrolithiasis, and often, renal phosphate wasting and impaired urinary acidification, resulting in rickets [70].

*Iatrogenic phosphate wasting* may be caused by exposure to certain chemotherapies such as cisplatin and ifosfamide, and other drugs (e.g., tenofovir, protease inhibitors) [71].

## General diagnostic approach

The diagnosis of rickets is based on typical clinical symptoms (e.g., widened wrists, frontal bossing, leg deformities, waddling gait, muscle weakness, and growth failure) and radiological findings (e.g., metaphyseal fraying and widening of growth plates) in the presence of elevated serum ALP levels (Fig. 6). The biochemical workup is based on assessment of serum phosphate, (ionized) calcium, creatinine, bicarbonate, ALP**,** PTH, 25(OH)D, 1,25(OH)_2_D, plasma FGF23 (if available) and an assessment of the renal tubular function (Table 4) [1, 7, 25].

**Table 4:** Biochemical workup in rickets

Serum/plasma	• Phosphate (Pi), calcium, ionized calcium, albumin • Creatinine, bicarbonate • Alkaline phosphatase (ALP) • Alanine transaminase (ALT) • Aspartate transaminase (AST) • Bone specific ALP (in cases of elevated ALT/AST) • Parathyroid hormone (PTH) • 25(OH)D, and 1,25(OH)_2_D • Intact and/or c-terminal fibroblast growth factor 23 (FGF23)
Spot urine	• Dipstick: glucose, protein, pH • Potassium, sodium, calcium, phosphate, creatinine, glucose, amino-acids • ß2-microglobuline (or other low molecular weight proteins)
Calculations	• Estimated glomerular filtration rate (GFR) [96] • Urine: calcium/ creatinine ratio • Urine: phosphate/ creatinine ratio • Tubular maximum reabsorption of Pi per GFR (TmP/GFR)^a^ • Fractional tubular reabsorption of Pi (TRP)^a^

^a^Calculations are given in Table 5

The differential diagnosis of rickets can be straightforward, e.g., an infant presenting with typical clinical symptoms and a history of low calcium intake and lack of vitamin D prophylaxis suggests nutritional rickets, but can be tricky in a child with a rare case of hereditary rickets, especially when presenting with mild symptoms and concomitant decreased vitamin D levels, which is not uncommon. As outlined above, hypophosphatemia is the denominator of all forms of rickets, except metabolic acidosis and CKD-associated rickets, which must be excluded before using the diagnostic algorithm shown in Fig. 6. This approach was originally proposed by Penido and Alon [3] and is based on the underlying mechanisms of hypophosphatemia, i.e., i) elevated levels of the phosphaturic hormone PTH, ii) inadequate intestinal phosphate absorption, and iii) renal phosphate wasting. The latter can either be due to increased activity of the phosphaturic hormone FGF23 or primary/acquired tubular defects. Further details of the underlying entities are given in Table 1. The use of the diagnostic algorithm based on patient history, clinical presentation, laboratory and imaging studies, as well as its pitfalls, are outlined in the following section.

## Medical history

A detailed medical history, including dietary intake and medication, as well as family history, is essential in order to establish the mode of inheritance. The recommended dietary calcium intake in order to prevent rickets amounts to 200 and 260 mg/day in infants 0–6 and 6–12 months of age, respectively [28]. Thereafter, an intake above 500 mg/day is recommended, and an intake of less than 300 mg is considered to be deficient (Table 5). However, there is no clear cut-off at which low calcium intake results in nutritional rickets, as this is often due to a combination of low calcium intake and vitamin D deficiency, which are both associated with the various risk factors given in Table 2**,** which should also be considered [26, 28–30]. Breast milk-fed infants of vitamin D deficient mothers are most prone to hypocalcemic complications such as poor feeding, irritablitiy, and seizures if unsupplemented. Older patients may complain of limb pain, muscle spasms, and fatigue (Table 3).

**Table 5:** Reference values for serum and urine biomarkers used for assessment of rickets and recommended dietary calcium intake

Age and/or sex specific values														
	**iCa** mmol/L [97, 98]	**Ca** mmol/L [97, 98]		**Pi** mmol/L [99, 100]		**TmP/GFR** mmol/L [75, 76, 101]			**ALP** U/L [81, 102]		**U**_**Ca/Crea**_ mol/mol (mg/mg) [103, 104]	**U**_**Pi/Crea**_ mol/mol (mg/mg) [103]		**1,25(OH)**_**2**_**D** pmol/L (ng/L) [83, 105]
**0–5 m**	1.22–1.40	2.17–2.82		1.25–2.50	**0–5 m**	1.02–2.0	**0–15 days**		90–273	**0.1– < 1 y**	0.09–2.2 (0.03–0.81)	1.2–19.0 (0.34–5.24)	**0– < 1 y**	77–471 (31–188)
**6–12 m**	1.20–1.40	2.17–2.75		1.15–2.15	**6–11 m**	1.13–1.88	**15–30 days**		134–518	**1– < 3 y**	0.07–1.5 (0.03–0.56)	1.2–14.0 (0.34–3.95)	**1– < 3 y**	113–363 (45–145)
**1–5 y**	1.22–1.32	2.35–2.70		1.05–1.95	**1–5 y**	1.05–1.78	**1– < 10 y**		156–369	**3– < 5 y**	0.05–1.1 (0.02–0.41)	1.2–12.0 (0.33–3.13)	**3 – 19 y**	108–246 (43–98)
**6–12 y**	1.15–1.32	2.35–2.57		1.00–1.80	**6–12 y**	0.97–1.64	**10– < 13 y**		141–460	**5– < 7 y**	0.04–0.8 (0.01–0.30)	1.2–5.0 (0.33–1.49)	**Adults**	75–200 (30–80)
**13–15 y**	1.21–1.30	2.20–2.55		0.95–1.65	**13–15 y**	0.91–1.68	**13– < 15 y**		F: 62–280 M: 127–517	**7– < 10 y**	0.04–0.7 (0.01–0.25)	1.2–3.6 (0.32–0.97)		
**16–19 y**	1.21–1.30	2.20–2.55		0.85–1.60	**16–18 y**	0.84–1.23	**15– < 17** y		F: 54–128 M: 89–365	**10– < 14 y**	0.04–0.7 (0.01–0.24)	0.8.–3.2 (0.22–0.86)		
**Adults**	1.12–1.32	2.15–2.58		0.84–1.45	**Adults**	0.84–1.23	**17– < 19** y		F: 48–95 M: 59–164	**14– < 18 y**	0.04–0.7 (0.01–0.24)	0.8–3.2 (0.21–0.75)		
							**Adults**		F: 33–98 M: 43–115	**Adults**	< 0.57 (0.2)	n.a		
**Age-independent specific values**														
**TRP** % [106]	**Intact PTH** pmol/L [107]				**25(OH)D** nmol/L (ng/L) [28, 108]			**Calcium intake for all children > 12 months per day**^a^ mg/day [28]						
85–95	1.5–6.5		**Sufficient**		> 50 (20)			> 500						
			**Insufficient**		30–50 (12–20)			300–500						
			**Deficient**		< 30 (12)			< 300						

iCa: ionised calcium; Ca: calcium; Pi: phosphorus; ALP: alkaline phosphatase; U: urine; Crea: creatinine; PTH: parathyroid hormone; 25(OH)D: 25 hydroxy vitamin D (calcidiol); 1,25(OH)_2_D: 1,25-dihydroxyvitamin; m: months; y: years; M: males; F: females; n.a.: not available

^a^Calcium requirement of infants aged 0–6 and 6–12 months is 200 mg/day and 260 mg/day, respectively [28]

Conversion factor for calcium and ionized calcium: mmol/l 4.01 ×  = mg/dL; conversion factor for phosphate: mmol/L × 3.097 = mg/dL; conversion factor for intact PTH: pmol/L × 10 = ng/L. NOTE. SI Units × Conversion Factor = Metric Units

TRP and TmP/GFR are calculated by entering the fasting urine and plasma concentrations, in the same concentration units, into the following equations:

TRP = 1 – ((Up/Pp) × (Pcr/Ucr)); TmP/GFR = Pp – (Up/ Ucr) × Pcr [74, 77]

An online calculator is available at: https://gpn.de/service/tmp-gfr-calculator/

An elemental or hypoallergenic formula diet or parental nutrition may cause hypophosphatemic rickets [38, 39]. A history of gastrointestinal surgery may point to impaired dietary phosphate availability. Certain drugs, including valproate, cisplatin, ifosfamide, gentamycin, and excessive use of phosphate binders, may cause renal Fanconi syndrome or interfere with calcium/vitamin D metabolism or phosphate intake [68, 71]. In cases of symptoms such as polyuria, polydipsia, and failure to thrive, a workup for Fanconi syndrome should be initiated.

If a family member is affected, they should be investigated for disproportionate short stature, leg deformities, abnormalities of skull shape (frontal bossing), history of previous orthopedic operations, dental abscesses, periodontal disease, permanent tooth and hearing loss, suggesting XLH. These patients may also suffer from “bone pain” due to osteoarthritis, enthesopathy and pseudofractures. Due to its X-dominant inheritance, 50% of the offspring of affected females and all daughters, but not sons, of affected males will be affected. However, about 30% of XLH cases are due to de novo mutations. An autosomal, dominant inheritance suggests ADHR (Table 1), whereas an X-linked recessive inheritance suggests Lowe syndrome or Dent disease, often presenting with Fanconi syndrome. In a patient with unaffected parents, especially if they are consanguineous or, in the case of an affected sibling, several autosomal recessive disorders should be considered, including VDDR types 1A, 1B, 2A, and 2B, ARHR types 1 and 2, HHRH, hypophosphatemia, nephrocalcinosis, and nephropathic cystinosis.

## Physical investigation

A detailed examination, including assessment of height and sitting height, in order to detect a disproportionate short stature (short legs and normal trunk length), presence of limb deformities (genu varum and genu valgum), and thickened wrists and ankles (widened metaphysis), and enlarged costochondral junctions of the rib (rachitic rosary)) should be undertaken. The degree of limb deformities may be assessed by measurement of the intermalleolar and intercondylar distances [72]. A waddling gait may be related to muscle weakness and/or coxa vara. Tibial intorsion may present with an in-toed gait. The head should be assessed for abnormal shape with frontal bossing and dolichocephaly, craniotabes and large fontanelle in infants. A detailed neurological examination and fundoscopy should be undertaken in patients with signs suggesting craniosynostosis/Chiary type 1 malformations, including dolichocephaly, persistent headache and/or ataxia.

It must be pointed out that there is a considerable overlap of the clinical features of the various causes of calcipenic and phosphopenic rickets, making it usually impossible to render a specific diagnosis solely based on clinical examination. In general, clinical presentation may be more severe in patients with VDDR. Neuromuscular irritability and tetany during provocation tests (Chvostek and Trousseau signs) suggests hypocalcemia, which is noted in calcipenic but not in hypophosphatemic rickets (Fig. 7). Characteristic clinical features pointing to certain causes of rickets are given in Table 3.

## Laboratory investigations

### Serum phosphate

Serum Pi levels are decreased in most forms of rickets, and tend to be even more decreased in patients with phosphopenic rickets compared to calcipenic rickets, as the designations already suggest. In patients with calcipenic rickets, serum phosphate levels may initially remain in the lower normal range until PTH-related renal phosphate loss has resulted in demineralized bone, which makes maintenance of normal serum phosphate levels impossible. Serum Pi levels must always be interpreted together with other biochemical parameters, including PTH values and parameters of urinary phosphate handling (vide infra), which are strongly age-dependent, with highest values during the first months of life and a continuous decrease until adulthood (Table 5). Establishing a diagnosis of hypophosphatemia in patients with inherited forms of hypophosphatemic rickets, such as XLH, is hampered in early infancy as serum phosphate levels often remain in the lower normal range during the first 4–6 months [3]. It must be stressed that published pediatric reference values differ considerably and it is recommended that diagnosis of hypophosphatemia is based on locally established pediatric reference values, if available [73]. In Table 5, we present one widely used data set. Repeated measurements may be required to confirm hypophosphatemia and it should be taken into account that pediatric patients may show lower serum phosphate concentrations compared to healthy pediatric volunteers, which hampers the interpretation of reduced serum phosphate levels in these patients. Finally, due to the diurnal fluctuations of serum phosphate levels with lowest levels in the morning, fasting state, and highest levels in the afternoon and evening, it is recommended to establish the diagnosis of hypophosphatemia in the morning fasting state [7, 36].

### Renal phosphate handling

Determination of tubular maximum reabsorption of Pi per glomerular filtration rate (TmP/GFR) using a second morning spot urine and serum sample taken at the same time allows for proof of renal phosphate wasting [74–77]. Both circulating levels of Pi and TmP/GFR are highest in infants and young children, and constantly decline thereafter until adulthood. Three methods (two formula and one nomogram-based) are available to estimate TmP/GFR and it is important to employ the appropriate reference data using the same methodology [77]. A formula proposed by Brodehl et al. to calculate TmP/GFR in children and adolescents, which is reliable in the fasting and non-fasting state, age-related reference values, and a link to an online calculator are presented in Table 5. The nomogram from Walton and Bijvoet was originally established in adults [78]. Its use is not recommended in children, as it results in a slight overestimation of TmP/GFR when used in this population, as compared to the formula provided by Brodehl et al. [77]. Finally, the formula and respective reference values provided by Payne et al. may be employed [79]. The calculation of fractional tubular reabsorption of phosphate (TRP) is not reliable for assessing renal phosphate handling, as it does not account for the amount of filtered phosphate. In the setting of very low serum phosphate concentrations, the remaining capacity of the proximal renal tubules may still be enough to maintain a normal TRP, whereas TmP/GFR is already clearly reduced.

It is necessary to mention that the assessment of TmP/GFR does not allow for discrimination between calcipenic or phosphopenic rickets, as in both entities renal phosphate wasting, and thus reduced TmP/GFR is present, either due to increased PTH (calcipenic rickets), or FGF23 activity or inherited/ acquired impairment of tubular phosphate transporters (phosphopenic rickets) (Table 1).

There is one important pitfall when evaluating renal phosphate wasting by calculating TmP/GFR. In rare cases, when hypophosphatemia is due to decreased phosphate bioavailability or insufficient phosphate intake from the gut, TmP/GFR values may be “falsely” reduced (Table 1) [3]. This dilemma becomes clear when looking at the formulae given in Table 5. As TmP/GFR amounts to serum phosphate concentration minus a sum based on the ratios of urinary and serum phosphate and creatinine concentrations, it is per se always equal to or less than the serum phosphate concentration. Consequently, estimated TmP/GFR may be reduced in cases of dietary phosphate deficiency or impaired bioavailability, resulting in very low serum phosphate levels, even when correcting for filtered amounts of Pi. This holds true for all available methods [3, 74, 78, 79]. The clue to identifying this pitfall is a very low urine phosphate concentration, which can easily be assessed by calculating the urinary phosphate to creatinine ratio (U/_P/Crea_). In case of low U/_P/Crea_, TmP/GFR should only be assessed after raising serum and urine phosphate levels via phosphate supplementation [3]. This also explains why urinary phosphate levels (U/_P/Crea_) and not TmP/GFR was introduced into the diagnostic algorithm given in Fig. 6. Pediatric reference values for U/_P/Crea_ are given in Table 5.

### Urine analyses

A urine dipstick allows for identification of glucosuria and proteinuria, suggesting renal Fanconi syndrome. Urinary calcium to creatinine ratio (U_Ca/Crea_) should be assessed using a spot urine sample, related to age-specific reference values (Table 4), and should be confirmed by repeated measurement or a 24 h urine sample, in cases of abnormal values [7]. A urinary calcium excretion above 4 mg/kg per day indicates hypercalciuria. Patients with calcipenic rickets usually present with low U_Ca/Crea_, due to impaired vitamin D metabolism. Patients with FGF23-mediated renal phosphate wasting usually also show low U_Ca/Crea_ values due to diminished 1,25 vitamin D synthesis, whereas most patients with primary renal tubular phosphate wasting show hypercalciuria due to elevated 1,25(OH)_2_D synthesis (e.g., HHRH, IIH, cystinosis, Dent disease), which can be associated with nephrocalcinosis/nephrolithiasis (Table 1). Therefore, a patient with a presumed diagnosis of XLH presenting with hypercalciuria should never be started on conventional treatment (phosphate supplements and active vitamin D) or burosumab—an FGF23 antibody—as this may promote progressive nephrocalcinosis. Instead, a diagnostic workup for other causes of rickets, as outlined above, should be undertaken. Finally, urine amino acids and low molecular weight proteins should be evaluated to detect Fanconi syndrome.

### Serum calcium

Serum calcium levels are only minimally age-dependent, with higher levels in young children (Table 5). Ionized calcium, or—if not available—albumin-corrected calcium levels should be used in patients with hypoalbuminemia. Patients with calcipenic rickets usually present with low calcium levels, but serum levels may be in the lower normal range in the early stages, when PTH-driven calcium release from bone can compensate for calcium deficiency. By contrast, serum calcium levels in treatment-naïve patients with phosphopenic rickets are usually in the normal range (Table 1).

### Serum alkaline phosphatase

Serum ALP levels serve as a marker of osteoblast activity and are generally elevated in all forms of rickets [1, 80]. In children, 80–90% of total ALP has a bone origin. Therefore, total ALP may be used instead of bone-specific ALP in pediatric patients after exclusion of liver disease, which includes the evaluation of liver enzymes [7]. ALP levels show a tetraphasic course, with highest serum concentrations in infancy and puberty and troughs at mid-childhood and post-puberty [81]. Therefore, ALP levels must always be interpreted in comparison with age- and sex-related normative values. Unfortunately, these differ markedly depending on the assay used. In Table 5, we show recent international pediatric reference level values, which may be used when local normal values are not available. Pediatric reference values for bone-specific ALP are also available [82].

Elevated serum ALP levels confirm the diagnosis of rickets in patients with clinical and radiological signs of rickets. By contrast, normal/reduced ALP levels are seen in Blount’s disease, metaphyseal dysplasia, and hypophosphatasia, which may mimic rickets.

ALP levels are usually highly elevated in treatment-naïve patients with calcipenic rickets (up to tenfold upper normal limit (UNL) or more), and moderately elevated (1 to 3 times UNL) in those with phosphopenic rickets (Table 1). A study comparing age and sex-related z-scores for total ALP levels in treatment-naïve patients with different types of rickets revealed mean values of 11.2 ± 2.6 (VDRR), 7.1 ± 3.8 (nutritional rickets), and 4.2 ± 1.6 (hypophosphatemic rickets), respectively (p < 0.001 between groups) [80]. However, there was a considerable overlap between groups. Finally, circulating ALP is a useful marker in disease monitoring.

### Parathyroid hormone

Major stimuli for the synthesis of PTH in the parathyroid glands are hypocalcemia, low 1,25(OH)_2_D levels, and hyperphosphatemia, whereas hypercalcemia and hypophosphatemia suppress PTH levels (Fig. 4) [16]. PTH levels are markedly increased in untreated patients with calcipenic rickets in order to maintain normal serum calcium levels (Table 1). By contrast, PTH levels are usually in the normal range in patients with phosphopenic rickets, but can be slightly elevated in patients with FGF23-driven phosphopenic rickets, as FGF23 suppresses 1,25(OH)_2_D levels, which in turn stimulates PTH secretion in the parathyroid glands. PTH levels are often suppressed in patients with HHRH, due to 1,25(OH)_2_D excess. Finally, PTH levels are expected to be markedly elevated in children presenting with rickets due to CKD, which is usually associated with elevated serum phosphate and creatinine levels. Monitoring of PTH is an excellent marker for the response to therapy in patients with calcipenic rickets.

### Vitamin D levels

Serum levels of 25(OH)D are a reliable marker of vitamin D status in a child. Vitamin D sufficiency is defined by 25(OH)D levels above 50 nmol/l, insufficiency by levels between 30 and 50 nmol/l, and deficiency by levels below 30 nmol/l (Table 5) [28]. Serum 25(OH)D levels are usually normal in patients with phosphopenic rickets. However, vitamin D insufficiency is frequently observed in the general population. In addition, normal 25(OH)D levels do not exclude nutritional rickets as nutritional rickets may also be related to reduced dietary calcium intake. In addition, reduced 25(OH)D levels are also observed in VDDR types 1B and 3 (Table 1). Therefore, 25(OH)D levels must be carefully interpreted together with a history of dietary intake (vide supra) and other biochemical parameters including 1,25(OH)_2_D levels (vide infra).

If the proposed diagnostic algorithm is followed, 25(OH)D values should be assessed in patients presenting with calcipenic rickets, as suggested by elevated PTH levels (Fig. 6). According to a global consensus recommendation on prevention and management of nutritional rickets, a serum 25(OH)D level below 30 nmol/L (12 ng/ml) supports the diagnosis of nutritional rickets due to vitamin D deficiency [28]. The duration of vitamin D deficiency, dietary calcium intake (vide supra), the patient’s growth rate and other risk factors (Table 2) must also be taken into account. If a patient with the presumed diagnosis of nutritional rickets does not respond to treatment with native vitamin D and calcium the diagnosis must be reconsidered, including other causes of low 25(OH)D levels (VDDR types 1B and 3).

Serum levels of 1,25(OH)_2_D vary largely depending on the methodology used, and local pediatric reference values may not be available. Pediatric reference values using a fully automated chemiluminescence immunoassay were published by the Canadian Laboratory Initiative in Pediatric Reference Intervals (CALIPER) [83]. Serum levels of 1,25(OH)_2_D were age-dependent with higher levels in infancy and stable levels after the age of 3 years, which is comparable to those in adults (Table 5).

Increased 1,25(OH)_2_D levels in patients with calcipenic rickets suggest a diagnosis of calcium deficiency or VDR defects, i.e., VDDR types 2A and 2B, whereas reduced 1,25(OH)_2_D levels suggests VDDR types 1A, 1B and 3. Diagnosis of the latter two is further supported by low 25(OH)D levels (Table 1, Fig. 5).

In patients with FGF23-mediated rickets, 1,25(OH)_2_D levels are usually low or inappropriately normal in the setting of hypophosphatemia. By contrast, 1,25(OH)_2_D levels are usually increased in patients with an inherited renal tubular transporter (NaPi2a and 2C) defect. Patients with nephropathic cystinosis may show normal or reduced 1,25(OH)_2_D levels, depending on the CKD stage (Table 1) [84].

### Fibroblast growth factor 23

The measurement of FGF23 levels is helpful in the diagnostic workup of phosphopenic rickets in order to differentiate between FGF23-mediated and other forms (Fig. 6, Table 1) [3, 7, 36, 85, 86]. Several ELISA assays for biological, active, intact FGF23 (Immunotopics, San Clemente, CA, USA; Kainos, Tokyo, Japan; Millipore, Bedford, MA), an automated, intact method (Diasorin liaison, Saluggia, Italy) and c-terminal FGF23 (Immunotopics; Biomedica ELISA, Wien, Austria; Quidel) are available. Use of plasma EDTA is recommended for most assays. FGF23-concentrations decline when centrifugation is delayed (> 1 h) [87]. Therefore, prompt centrifugation is recommended to avoid falsely normal levels. Age-related reference values for intact [84] and c-terminal FGF23 [82] are available, but vary considerably according to the assay used. It is therefore recommended that the results in an individual patient should always be compared with the reference value obtained by the same assay. Pi intake and vitamin D therapy strongly enhance serum FGF23 levels. FGF23 levels are 2–threefold higher in XLH patients receiving conventional treatment with oral phosphate and active vitamin D, compared to untreated patients [88, 89]. Therefore, FGF23 levels are most informative in untreated patients. In patients have already started on conventional treatment, fasting Pi and FGF23 samples should ideally be collected 1 or 2 weeks after discontinuation of treatment. FGF23 levels are also not informative in patients on burosumab treatment, as this interferes with the FGF23 assay. Indeed, because burosumab binds FGF23 in the circulation, this may result in markedly elevated intact and c-terminal FGF23 concentrations, irrespective of the assay used [90]. Finally, high normal FGF23 levels in the setting of hypophosphatemia should be interpreted as inappropriately normal and therefore do not exclude FGF23-driven hypophosphatemia [7].

Endo et al. evaluated the diagnostic value of intact FGF23 levels, using the Kainos assay in two studies in pediatric and adult Japanese patients with FGF23-mediated hypophosphatemia, including TIO, XLH, ARHR, ADHR, McCune-Albright syndrome/fibrous dysplasia in comparison to patients with non-FGF23-mediated forms of rickets (e.g., nutritional rickets, VDDR, Fanconi syndrome) [91, 92]. FGF23 levels were above the UNL (50 pg/ml) in most patients of the former group and were undetectable or below 23.9 pg/ml in the latter group. Mean FGF23 levels were significantly higher in TIO patients compared to those with FGF23-mediated rickets due to genetic defects, but varied widely in both groups. The combination of hypophosphatemia and FGF23 levels above 30 pg/ml allows for identification of all patients with TIO and genetic hypophosphatemia and excludes all patients with other forms of rickets. From these results, they proposed a cut-off level of 30 pg/ml to confirm FGF23-mediated hypophosphatemia in children and adults when using the Kainos assay [85].

As the degree of elevated FGF23 does not allow for discrimination between the various forms of FGF23-mediated rickets, final diagnosis may require additional genetic testing, especially in cases of negative family history. Bearing in mind the uncertainties of FGF23-assessment, one may also decide upon genetic testing if results are readily available. However, in cases of negative genetic testing for inherited forms of hypophosphatemic rickets, non-genetic forms such as TIO must be considered.

## Apparative diagnostics

### Radiographs

Radiological changes in rickets are best assessed on radiography at the growth plates of rapidly growing bones, i.e., the distal ulna, metaphyses around the knees and ankles. Early signs include increased height of the physis, widening of the epiphyseal plate and loss of definition in the zone of provisional calcification at the epiphyseal/metaphyseal interface (Figs. 2 and 3) [1, 7]. Later on, a progressive disorganization of the growth plate with cupping, splaying, formation of cortical spurs and stippling, as well as a delay in the appearance of the epiphyseal bone centers is noted. Deformities of the weight-bearing long bones may be present. Finally, Milkman pseudofractures, characterized by pathological fractures and Looser's zones, narrow radiolucent lines 2 to 5 mm in width with sclerotic borders, may be present in adolescents and adults suffering from XLH.

The severity of rickets in XLH patients was monitored in clinical trials using the Rickets Severity Score (RSS), a quantitative scale originally created for assessment of nutritional rickets by Tom Thacher [93, 94]. RSS is based on the degree of metaphyseal fraying and concavity, and the proportion of the growth plate affected at the wrist and knee. It is important to note that radiography of XLH patients shows the metaphyseal signs seen in common rickets, but lacks signs of bone resorption and does not usually show radiolucency. This explains why the Thacher score in patients with XLH is usually graded below 3 on a scale of 10 [93]. Unfortunately, it requires simultaneous radiography of the wrist and knee and is therefore associated with higher X-ray exposure, compared to routine radiography of one joint, which hampers its use in clinical practice.

### Kidney ultrasound

The presence of nephrocalcinosis or nephrolithiasis in a patient prior to initiation of therapy, suggests diseases associated with hypercalciuria such as HHRH (*NaPi 2C* defects), Dent disease 1(*CLCN5* defect) or hypophosphatemia and nephrocalcinosis (*NaPi2a* defects). Nephrocalcinosis may also be noted in children with nephropathic cystinosis and was observed in 30–70% of XLH patients on long-term conventional treatment. Diagnosis of FGF23-mediated phosphopenic rickets, including XLH, should always be questioned in a treatment-naïve patient showing nephrocalcinosis.

### Cardiovascular investigations

Infants with nutritional rickets should undergo a heart echo- and electrocardiogram evaluation, in order to detect dilated cardiomyopathy (heart failure, arrhythmia) [29]. Left ventricular hypertrophy and/or hypertension have rarely been reported in XLH patients on long-term conventional treatment. Assessment for these changes may not be required at the initial diagnosis workup [7].

### Diagnostic workup for TIO

Children manifesting with hypophosphatemic rickets after early childhood, associated with elevated FGF23 levels, negative family history, and/or negative genetic tests for hereditary causes should be candidates for a TIO diagnostic workup [60]. The biochemical changes resemble those seen in XLH. Localization of these, usually, very small and slow-growing tumors is challenging. They may be located anywhere in soft tissue or the skeleton and are more likely seen in craniofacial bones or extremities. A palpable mass can rarely be identified. Therefore, full-body imaging techniques, including ^68^GA-DOTA-based PET/CT scans and Technecium 99 m octreotide with single-photon emission computed tomography (octro-SPECT), are suggested for tumor localization, which should be followed by histological confirmation. A comprehensive review on the optimal diagnostic approach was recently provided by Florenzano et al. [60].

## Genetic studies

The diagnostic measures outlined above may not allow for confirmation of the exact underlying cause in patients with inherited forms of rickets, especially in cases of negative family history or unusual clinical presentation. In addition, burosumab, a humanized FGF23 antibody, has only been approved in patients with XLH and TIO, but not in other forms of FGF23-driven phosphopenic rickets. Therefore, genetic confirmation of the diagnosis is generally recommended, unless a non-genetic cause of rickets can be proven, or in cases of a positive family history and clear clinical presentation (Fig. 6) [1, 7]. *PHEX* mutations can be found in 87% of familial cases and 72% of sporadic cases of clinically and laboratory classified XLH [40, 95].

### Key summary points

- The diagnosis of rickets is based on the presence of typical clinical symptoms and radiological findings on X-rays of the wrist or knee, showing widening of the growth plates and metaphyseal fraying and in conjunction with elevated serum levels of alkaline phosphatase.
- Nutritional rickets, due to vitamin D deficiency and/or dietary calcium deficiency, is the most common cause of rickets.
- Hereditary causes of rickets are due to mutations in genes involved in vitamin D metabolism or action, renal phosphate reabsorption, or synthesis or degradation of the phosphaturic hormone fibroblast growth factor 23.
- There is a substantial overlap of the clinical features between the various entities, requiring a thorough workup by biochemical analyses and, if necessary, genetic tests.

## Multiple choice questions

1. Which of the following statements is true?
  Clinical signs of rickets are:
  - Blue sclerae and an increased fracture rate.
  - Early childhood caries.
  - Progressive deformities of the lower extremities.
  - Shortened upper extremities.
  - Premature closure of cranial sutures.
2. Which of the following statements is true?
  - The daily vitamin D requirement is mainly met from animal products.
  - Vitamin D promotes the renal excretion of calcium and phosphate.
  - The most metabolically effective vitamin D metabolite is 25-hydroxy vitamin D.
  - The liver and kidneys are important sites for vitamin D metabolite synthesis.
  - In Europe, endogenous vitamin D synthesis is strongly reduced from April to September.
3. Which of the following statements is true?
  - Calcipenic rickets is usually associated with normal serum phosphate and parathyroid hormone levels.
  - 1,25(OH)_2_D levels are reduced in nutritional rickets.
  - Increased renal calcium excretion is a common finding in calcipenic rickets
  - Alkaline phosphatase levels are usually less increased in calcipenic rickets compared to hypophosphatemic rickets.
  - Reduced calcium intake and/or low 25-OHD levels may cause calcipenic rickets.
4. Which of the following statements is true?
  - Hypophosphatemic rickets results, in most cases, from a diminished phosphate intake.
  - Hypophosphatemic rickets can be caused by elevated levels of fibroblast growth factor 23 or primary renal phosphate wasting.
  - Hypophosphatemic rickets mainly affects adolescents rather than young children.
  - In contrast to calcipenic rickets, hypophosphatemic rickets is associated with impaired apoptosis of hypertrophic chondrocytes, resulting in widening of the growth plates in bones.
  - Hypophosphatemic rickets is usually associated with increased levels of parathyroid hormone, resulting in decreased serum phosphate concentrations.
5. Which of the following statements is true?
  - Hypophosphatemic rickets is most commonly inherited as an X-linked dominant disorder.
  - Hypophophatemic rickets is most commonly inherited as an autosomal dominant disorder.
  - In XLH, *PHEX* gene mutations can be detected in approx. 50 percent of cases.
  - In the case of a father affected by XLH, the daughters and sons have a 50% risk of XLH.
  - Hypophosphatemic rickets is most commonly inherited as an autosomal recessive disorder.
